# The Role of Oxidative Stress in the Pathogenesis of Childhood Asthma: A Comprehensive Review

**DOI:** 10.3390/children12091110

**Published:** 2025-08-23

**Authors:** Despoina Koumpagioti, Margarita Dimitroglou, Barbara Mpoutopoulou, Dafni Moriki, Konstantinos Douros

**Affiliations:** 1 Department of Nursing, University of West Attica, 12243 Athens, Greece; 2 Respiratory and Allergy Unit, 3rd Pediatric Department, General University Hospital “Attikon”, National and Kapodistrian University of Athens, 12462 Athens, Greece; margie-15@hotmail.com (M.D.); kdouros@med.uoa.gr (K.D.); 3 Department of Nursing, National and Kapodistrian University of Athens, 11527 Athens, Greece; bmpoutopoulou@gmail.com

**Keywords:** oxidative stress, reactive oxygen species, reactive nitrogen species, biomarkers, antioxidants, airway inflammation, therapy, childhood asthma

## Abstract

**Highlights:**

**What are the main findings?**

- Oxidative stress is a central driver of childhood asthma—linking environmental and endogenous ROS/RNS to airway inflammation, epithelial damage and remodeling, and reduced corticosteroid responsiveness.
- Redox-sensitive biomarkers (exhaled 8-isoprostane, H_2_O_2_, FeNO) rise with disease activity; susceptibility is further shaped by prenatal/early-life exposures (e.g., PM_2.5_, tobacco smoke) and antioxidant-gene variants (GSTP1, CAT).

**What is the implication of the main finding?**

- Incorporating oxidative-stress assessment and redox biomarkers into monitoring could help identify high-risk children and anticipate exacerbations.
- Adjunct antioxidant and lifestyle strategies (Mediterranean-style diet, regular exercise) and emerging Nrf2/GSH-targeted therapies may improve control and enable personalized care based on redox profiles and genetic susceptibility.

**Abstract:**

This review aims to provide a comprehensive overview of how oxidative stress drives inflammation, structural remodeling, and clinical expression of childhood asthma, while critically appraising emerging redox-sensitive biomarkers and antioxidant-focused preventive and therapeutic strategies. Oxidative stress arises when reactive oxygen species (ROS) and reactive nitrogen species (RNS) outpace airway defenses. This surplus provokes airway inflammation: ROS/RNS activate nuclear factor kappa-B (NF-κB) and activator protein-1 (AP-1), recruit eosinophils and neutrophils, and amplify type-2 cytokines. Normally, an antioxidant network—glutathione (GSH), enzymes such as catalase (CAT) and superoxide dismutase (SOD), and nuclear factor erythroid 2-related factor 2 (Nrf2)—maintains redox balance. Prenatal and early exposure to fine particulate matter <2.5 micrometers (µm) (PM_2.5_), aeroallergens, and tobacco smoke, together with polymorphisms in glutathione S-transferase P1 (GSTP1) and CAT, overwhelm these defenses, driving epithelial damage, airway remodeling, and corticosteroid resistance—the core of childhood asthma pathogenesis. Clinically, biomarkers such as exhaled 8-isoprostane, hydrogen peroxide (H_2_O_2_), and fractional exhaled nitric oxide (FeNO) surge during exacerbations and predict relapses. Therapeutic avenues include Mediterranean-style diet, regular aerobic exercise, pharmacological Nrf2 activators, GSH precursors, and mitochondria-targeted antioxidants; early trials report improved lung function and fewer attacks. Ongoing translational research remains imperative to substantiate these approaches and to enable the personalization of therapy through individual redox status and genetic susceptibility, ultimately transforming the care and prognosis of pediatric asthma.

## 1. Introduction

Asthma is “a heterogeneous disease, usually characterized by chronic airway inflammation”. It is defined by a “history of respiratory symptoms—wheeze, shortness of breath, chest tightness and cough—that vary over time and in intensity, together with variable expiratory airflow”, according to the 2025 Global Initiative for Asthma (GINA) Strategy Report. These hallmark symptoms typically wax and wane, may be triggered by exercise, allergens, viral infections, or weather changes, and can resolve spontaneously or with appropriate treatment, setting asthma apart from other chronic respiratory disorders [1]. In 2021, the Centers for Disease Control and Prevention (CDC) national surveillance revealed that approximately 25 million people, 7.7% of the entire U.S. population, were living with current asthma, and 39.4% of them experienced at least one asthma attack during the preceding year. Nested within that overall burden are 4.7 million children, representing 6.5% of everyone under 18; nearly two-in-five of these children (38.7%) had an asthma attack in the same period [2].

Recent research has focused on elucidating the molecular mechanisms that drive the pathogenesis and progression of asthma, spotlighting oxidative stress as a pivotal contributing factor. A review demonstrated that children with asthma have elevated levels of reactive oxygen species (ROS), reactive nitrogen species (RNS), and oxidative stress biomarkers compared to healthy peers [3]. A self-reinforcing loop perpetuates this elevation. Environmental stimuli, such as allergens, air pollutants, and tobacco smoke, initiate oxidative reactions that drive inflammation. This inflammation, in turn, recruits eosinophils, neutrophils, and macrophages—cells that are themselves major producers of ROS, thereby amplifying oxidative stress even further [4]. Thus, both endogenous inflammatory cells and environmental stimuli heighten ROS/RNS in pediatric airways, correlating with more frequent attacks [3]. A panel study found that nasal malondialdehyde (MDA) mediated up to 14–57% of the symptom worsening provoked by colder temperatures in children with asthma [5].

While oxidative stress drives flare-ups in children with established asthma, growing evidence suggests it can be programmed before birth. A birth-cohort study links maternal exposure to traffic-related nitrogen dioxide (NO_2_) and fine particulate matter smaller than 2.5 micrometers (PM_2.5_) with higher fetal oxidative stress markers and smaller infant lung volumes, especially in babies carrying antioxidant-gene variants that weaken defenses [6]. Genetics further tips the scales. Gene polymorphisms in glutathione s-transferase Pi-1 (GSTP1) and catalase (CAT) can modulate the risk of childhood asthma, with certain variants linked to higher risk and others to a protective effect [7].

Downstream of these prenatal influences, dysbiosis of the airway and gut microbiomes perpetuates the same vicious cycle. Pollutant-driven shifts in microbial metabolites boost mucosal ROS/RNS production, fueling type-2 inflammation and diminishing steroid responsiveness [8]. Climate-related hazards layer on additional oxidative pressure. Wildfire smoke, for instance, delivers particulate-bound free radicals and is already linked to spikes in emergency department (ED) visits for asthma exacerbations [9].

Therapeutic strategies to prevent oxidant–antioxidant imbalance in childhood asthma include limiting environmental oxidant exposure, fortifying diets with antioxidants, employing redox-balancing therapies, and maintaining regular physical activity [10]. This review aims to provide a comprehensive overview of how oxidative stress drives inflammation, structural remodeling, and clinical expression of childhood asthma, while critically appraising emerging redox-sensitive biomarkers and antioxidant-focused preventive and therapeutic strategies.

## 2. Materials and Methods

This comprehensive review was conducted through a structured search of peer-reviewed literature in PubMed, Scopus, and Cochrane databases, covering studies published from January 2000 to May 2025. The search strategy included the terms: (“oxidative stress” OR “reactive oxygen species” OR “ROS” OR “RNS”) AND (“childhood asthma” OR “pediatric asthma”) AND (“biomarkers” OR “antioxidants” OR “airway inflammation” OR “therapy” OR “treatment” OR “intervention”). Two reviewers independently screened titles and abstracts for relevance, followed by a full-text review of selected articles. Discrepancies in study selection or interpretation were resolved through consensus discussion. Inclusion criteria comprised original research and review articles addressing oxidative stress-related mechanisms, biomarkers, or therapeutic strategies in asthma. While the primary focus was on childhood asthma, studies involving adult or mixed-age populations, as well as animal studies, were included if they provided mechanistic insights relevant to the review’s aim. Non-English articles and studies lacking accessible full texts were excluded.

**Figure 1:**
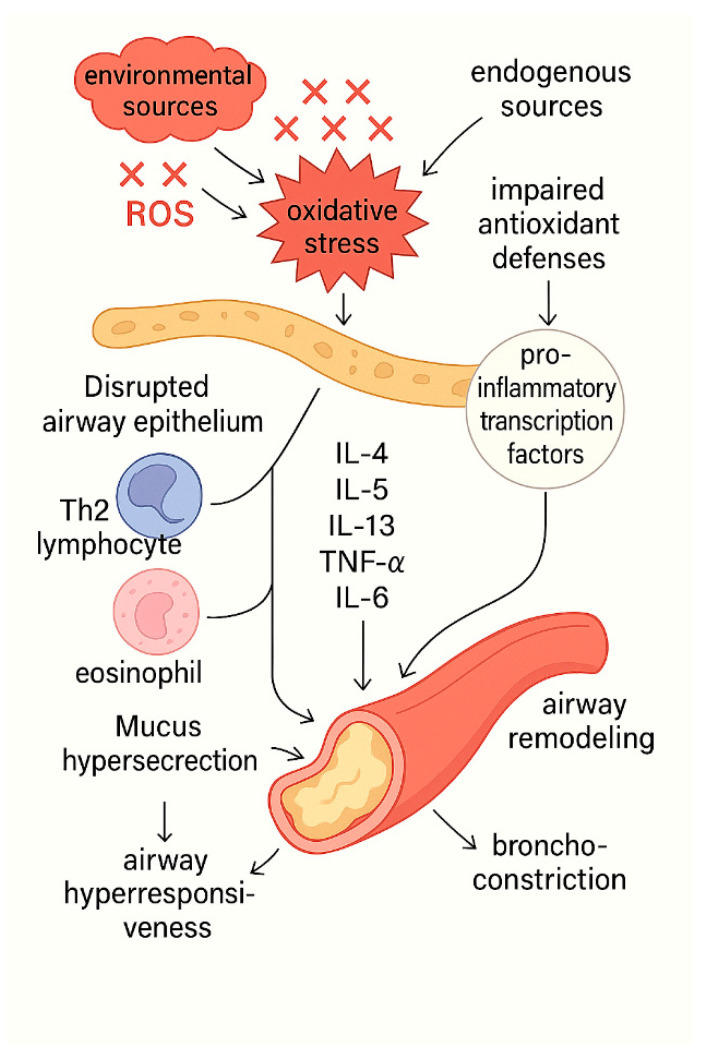
The central role of oxidative stress in asthma pathogenesis. ROS derived from both environmental and endogenous sources disrupt airway epithelial integrity and overwhelm antioxidant defense mechanisms, primarily by impairing Nrf2 signaling. This redox imbalance activates pro-inflammatory transcription factors, resulting in the release of cytokines such as IL-4, IL-5, IL-13, TNF-α, and IL-6. These mediators promote the recruitment and activation of Th2 lymphocytes and eosinophils, driving persistent airway inflammation and bronchoconstriction. The sustained inflammatory milieu leads to mucus hypersecretion, airway hyperresponsiveness, and structural airway remodeling, ultimately manifesting as the clinical features of asthma.

A generative artificial intelligence (GenAI) tool (ChatGPT 4o) was used in the creation of Figure 1 in this manuscript. These tools assisted in the visual design and illustration of the figure, based on the authors’ input and supervision. The authors reviewed and verified the final output to ensure its accuracy and appropriateness.

## 3. Oxidative Stress, the Role of ROS and RNS

Oxidative stress arises when the body’s antioxidant defenses can no longer counterbalance the surge of pro-oxidants, chiefly ROS and RNS. Inside the body (endogenous sources of ROS), these reactive molecules spring from numerous sources: intracellular organelles such as mitochondria, peroxisomes and the endoplasmic reticulum; diverse enzyme systems including cytochrome P450s, nicotinamide adenine dinucleotide phosphate (NADPH) oxidases (e.g., NOX2, NOX4), nitric-oxide synthases, and xanthine oxidase; immune and structural cells—notably phagocytes, activated eosinophils and neutrophils, monocytes and macrophages, plus airway epithelial, smooth-muscle and endothelial cells; and additional chemical routes involving heme proteins or redox reactions with metal ions [4,11,12,13].

However, the overall oxidative burden is shaped not only by endogenous processes; the contribution of diverse exogenous factors is equally decisive. Exogenous sources of ROS are also abundant. They stem from radiation exposure, redox-active metal ions, lung injury from ischemia–reperfusion, environmental pollutants laden with inorganic particles such as silica, quartz, vehicle exhaust, asbestos, and tobacco smoke, and particularly medications (notably chemotherapeutic agents). Together with endogenous ROS, these external oxidants drive and aggravate many inflammatory disorders by steadily accumulating oxidative damage in key biomolecules [4]. Table 1 illustrates the main sources and effects of oxidative stress in asthma.

To clarify the biochemical basis of this damage, it is useful to examine the principal ROS and RNS species and their key reactions. Superoxide anion (O_2_•^−^)—generated mainly by NADPH oxidase, xanthine oxidase and related enzymes—acts as the primary ROS and seed for a cascade of secondary oxidants, including hydrogen peroxide (H_2_O_2_), hydroxyl radical (•OH), singlet oxygen (^1^O_2_), peroxyl (LOO•) and alkoxyl (LO•) radicals, lipid hydroperoxides (LOOH), peroxynitrite (ONOO^−^), hypochlorous acid (HOCl) and ozone (O_3_). Although O_2_•^−^ and H_2_O_2_ are only moderately reactive, they are pivotal precursors for more aggressive oxidants [14].

The RNS arise when nitric oxide (NO•), produced from L-arginine by nitric oxide synthase 2 (NOS2), reacts further; NO• can combine with O_2_•^−^ to yield the highly reactive ONOO^−^ [15]. Transition metals such as iron (Fe^2+^) and copper (Cu^+^) intensify oxidative injury; notably, Fe^2+^ catalyzes the Fenton reaction, converting H_2_O_2_ into the extremely reactive •OH radical [16].

## 4. Oxidative Stress-Induced Airway Inflammation

Oxidative inflammation within the airways is a key driver in the pathogenesis of wheezing-lung phenotypes and several pulmonary disorders, including asthma, bronchopulmonary dysplasia (BPD), and pulmonary fibrosis [17].

Roughly one-third of children experience wheeze at least once during their first three years of life [18]. The sound arises when airflow is impeded by mucus, bronchial smooth muscle constriction (bronchospasm), or swelling of the airway epithelium. Typical wheeze frequencies span 100–1000 Hz, with occasional harmonics above 1 kHz [19]. In infants, even minimal lumen narrowing can be sufficient to provoke wheezing; their compliant chest wall enhances intrathoracic pressure during expiration, promoting transient airway collapse and sound generation [20]. By contrast, in older children, recurrent wheezing is most commonly attributable to asthma [21]. While mechanical factors such as bronchospasm and mucus plugging generate the audible wheeze, oxidative stress significantly contributes to these processes by enhancing smooth muscle reactivity and disrupting epithelial integrity—thus bridging the molecular and mechanical pathways of airflow obstruction [22,23]. Thus, wheeze can be interpreted not only as a mechanical hallmark of airflow limitation but also as the clinical sound manifestation of underlying ROS-driven airway pathology.

Beyond these mechanical airflow constraints, oxidative mediators emerge as pivotal drivers of airway inflammation. The airway epithelium serves as a tight physical barrier against microbes and comprises goblet, ciliated, club, and basal cells, among others [24]. During inflammation, pro-inflammatory cytokines recruit leucocytes—neutrophils, eosinophils, macrophages, and other mononuclear cells—to the epithelium, where they generate ROS such as O_2_•^−^, H_2_O_2_, and •OH [25]. Neutrophil and macrophage myeloperoxidase (MPO), as well as eosinophil peroxidase (EPO), catalyze the formation of hypohalous acids, namely HOCl and hypobromous acid (HOBr). These acids are strongly oxidative, making them useful for pathogen killing, yet potentially damaging to host tissues when unchecked [26].

These oxidants do not act in isolation; they trigger transcriptional programs that shift the balance from antioxidant defense toward overt inflammation, as outlined below. At modest oxidant burdens, nuclear factor-erythroid-2-related factor 2 (Nrf2) is activated, inducing more than 200 antioxidant genes and tempering oxidative stress [27]. Sustained or excessive oxidant levels, however, switch on pro-inflammatory transcription factors. Nuclear factor-κB (NF-κB) and activator protein-1 (AP-1) drive the expression of tumor necrosis factor-α (TNF-α), interleukins-1β, -6, and -8 (IL-1β, IL-6, IL-8), as well as cyclo-oxygenase-2 (COX-2) and matrix metalloproteinases (MMPs) [28]. These mediators compromise epithelial integrity, provoking mucus hypersecretion, smooth-muscle contraction, vascular leakage, epithelial sloughing, bronchial hyper-responsiveness, bronchoconstriction, and edema. NF-κB activity hinges on IκB phosphorylation by IκB kinase. At the same time, AP-1 transcription is shaped by serum-response factors (SRFs) and ternary-complex factors (TCFs), with NF-κB also feeding into AP-1 activation [26,29].

As this inflammatory environment persists, it progressively initiates airway remodeling. Repeated epithelial injury and repair cycles lead to collagen deposition, proliferation of goblet and bronchial smooth-muscle cells, epithelial hyper-replication, peri-bronchial fibrosis, and thickening of the reticular basement membrane. These changes narrow and stiffen the airways, heighten hyperresponsiveness, and ultimately foster the wheezing-lung phenotype [30].

Finally, dysbiosis directly contributes to chronic inflammation in asthma by disrupting the gut–lung axis and altering immune responses. The imbalance of beneficial bacteria reduces the production of short-chain fatty acids (SCFAs), which normally have anti-inflammatory effects, leading to increased activation of pro-inflammatory immune cells, such as Th2 lymphocytes and eosinophils [8]. As a result, more inflammatory mediators and cytokines are released, fueling persistent airway inflammation. This chronic inflammatory environment enhances oxidative stress, further aggravating asthma symptoms [8].

Figure 1 depicts the central role of oxidative stress in asthma pathogenesis.

## 5. Antioxidant Defense Systems in the Airway

ROS and RNS are continuously generated in human tissues, and the body relies on a network of antioxidant defenses, both enzymatic and non-enzymatic, to preserve redox homeostasis. The front-line antioxidant enzymes are superoxide dismutase (SOD), CAT, and glutathione peroxidase (GPX), which together intercept most free-radical assaults [31]. Additional intracellular defenders include heme oxygenase-1 (HO-1) and a family of redox proteins—thioredoxins, peroxiredoxins, and glutaredoxins—that are particularly important in the lung [32,33]. Non-enzymatic antioxidants such as vitamin C, vitamin E, essential trace minerals, and the thiol glutathione (GSH) complement these enzymes by directly scavenging radicals or recycling oxidized compounds [34,35]. Table 2 presents the key antioxidant defenses in the airway.

SOD catalyzes the dismutation of O_2_•^−^ and ^1^O_2_ into H_2_O_2_ and molecular oxygen (O_2_), thereby preventing the formation of more toxic species. The resulting H_2_O_2_ is then detoxified: CAT, located mainly in peroxisomes, splits H_2_O_2_ into water and oxygen, whereas GPX, which is abundant in mitochondria that lack CAT, reduces H_2_O_2_ and converts lipid hydroperoxides to their corresponding alcohols [12].

In the airways, additional highly specialized antioxidant systems act at the epithelial surface. The respiratory-tract lining fluid (RTLF) is enriched in extracellular SOD (EcSOD). This matrix-bound isoform removes superoxide before it can react with nitric oxide to form the potent oxidant peroxynitrite [36]. A second extracellular enzyme, lactoperoxidase (LPO), uses thiocyanate (SCN^−^) as a substrate to convert H_2_O_2_ into the antimicrobial—and less damaging—hypothiocyanite ion (OSCN^−^), simultaneously consuming excess peroxide in the airway lumen [37].

The RTLF also contains high concentrations (millimolar range) of reduced GSH, the dominant redox buffer at the air–liquid interface, and appreciable levels of uric acid secreted by airway epithelial cells; together with vitamins C and E, albumin-bound thiols and dietary carotenoids, these small molecules establish a potent chemical barrier against inhaled oxidants [38,39].

Finally, airway cells mount an inducible response through the Kelch-like ECH-associated protein 1 (Keap1)–Nrf2 signaling axis. Oxidative modification of Keap1 cysteines liberates Nrf2, which then upregulates a broad battery of antioxidant-response genes, including HO-1, γ-glutamyl-cysteine ligase (GCL), GPX2, and EcSOD, thereby enhancing both intracellular and extracellular defenses [40]. Among the Nrf2 targets, peroxiredoxin-6 (PRDX6)—a bifunctional 1-Cys peroxiredoxin with GSH-peroxidase and phospholipase A_2_ activities—detoxifies phospholipid hydroperoxides and repairs oxidized surfactant phospholipids, providing a surfactant-specific layer of protection in the distal lung [41].

Taken together, the classical intracellular enzymes, the airway-surface enzyme systems, the pool of low-molecular-weight antioxidants, and the Nrf2-driven inducible network create a multilayered defense that neutralizes both endogenous and inhaled oxidants before they can inflict epithelial injury or drive airway inflammation.

**Table 2:** Key antioxidant defenses in the airways.

Antioxidant System	Mechanism/Location	Role in Asthma
Enzymatic	SOD, CAT, GPX, HO-1 [31,32,33]	Neutralize ROS/RNS, protect epithelium
Non-enzymatic	Vitamin C, E, α-tocopherol, GSH, uric acid [34,35,42]	Scavenge radicals, support enzymes
Inducible—Nrf2 axis	Nrf2, Keap1, PRDX6, HO-1 [41]	Upregulate antioxidant gene expression

SOD: superoxide dismutase, CAT: catalase, GPX: glutathione peroxidase, HO-1: heme oxygenase-1, GSH: glutathione, Keap1: Kelch-like ECH-associated protein 1, Nrf2: nuclear factor-erythroid-2-related factor 2, PRDX6: peroxiredoxin-6.

## 6. Oxidative Stress and Its Role in the Pathogenesis of Childhood Asthma

Asthma involves airway inflammation coupled with exaggerated airway hyperresponsiveness (AHR) [43]. Its persistent inflammation manifests in two immune phenotypes: Type-2-high and Type-2-low [44]. Type-2-high asthma, generally allergy-driven, shows elevated eosinophils in the airways, whereas Type-2-low asthma is typically non-allergic and characterized by neutrophilic airway inflammation [45,46].

The airway epithelium normally forms a sealed barrier that blocks inhaled particles, but recurrent oxidative stress and ROS disrupt its tight and adherens junctions, increasing epithelial permeability. This leakiness lets allergens breach the compromised layer and reach the submucosa, where they stimulate immune cells and intensify airway inflammation [23].

As a direct consequence, oxidatively activated epithelial cells engage the redox-sensitive transcription factors NF-κB and AP-1, which in turn upregulate a suite of type-2–skewing cytokines, including IL-4, IL-5, and IL-13, together with the epithelial alarmin thymic stromal lymphopoietin (TSLP), thereby amplifying local inflammation. This cytokine milieu drives adaptive immunity toward pathogenic T helper 2 (Th2) and, in more severe or steroid-insensitive states, Th17 responses, perpetuating airway inflammation and structural remodeling [47]. Downstream, the same cytokines reprogram airway smooth muscle calcium ion (Ca^2+^) signaling. Pro-inflammatory cytokines, including IL-13, TNF-α, and interferon-gamma (IFN-γ), markedly upregulate two Ca^2+^-mobilizing modules in airway smooth muscle: the CD38/cyclic ADP-ribose (c-ADPR)-ryanodine-receptor axis and transient receptor potential canonical 3 (TRPC3)-mediated Ca^2+^ influx. This cytokine-driven remodeling amplifies agonist-evoked intracellular Ca^2+^ transients, thereby increasing contractile force and producing the airway smooth muscle hyperresponsiveness that typifies asthma and related wheezing phenotypes [48].

In parallel, ROS attack membrane lipids, generating 4-hydroxynonenal (4-HNE) adducts that act as immunogenic neoantigens and intensify allergic sensitization by presenting previously unseen epitopes to the immune system [49]. A two-year cohort of newly hired animal-facility workers, along with parallel C3H/HeJ mouse experiments, demonstrated that higher baseline serum 4-HNE adducts, coupled with weaker HO-1/Nrf2 antioxidant capacity, predicted subsequent sensitization to novel aeroallergens. These findings highlighted that oxidative protein and lipid modification directly amplify the allergy-priming step of asthma pathogenesis [50].

Over time, chronic oxidative stress also invokes profibrotic pathways driven by transforming growth factor-beta 1 (TGF-β1) and MMP-12, boosting collagen and fibronectin deposition beneath the epithelium. Together with TGF-β_1_-induced goblet cell proliferation and continued matrix accumulation, this response produces sub-epithelial fibrosis, goblet cell hyperplasia, and progressive thickening of the reticular basement membrane. These changes are hallmarks of chronic asthmatic airways [51].

Consistent with the oxidative-stress mechanisms outlined above, clinical biomarker studies demonstrate that redox load is measurably elevated in children with asthma. Shahid et al. showed that exhaled-breath 8-isoprostane levels are significantly higher in steroid-naïve (9.3 ± 1.7 pg mL^−1^) and steroid-treated (6.7 ± 0.7 pg mL^−1^) children with asthma compared to healthy controls (3.8 ± 0.6 pg mL^−1^), supporting its value as a non-invasive marker of oxidative stress and a potential indicator of exacerbation risk. The biomarker demonstrated good sensitivity, being consistently elevated in asthmatic children relative to controls, and excellent reproducibility (intra-class correlation coefficient = 0.98); however, it lacked specificity, as 8-isoprostane was not correlated with other markers such as fractional exhaled nitric oxide (FeNO) (r = −0.02, not significant) or lung function (FEV_1_ % predicted) [52]. In parallel, Caffarelli et al. detected markedly greater H_2_O_2_ concentrations in exhaled breath condensate during acute exacerbations (median 0.273 µM) and, importantly, persistently raised levels one week after therapy (0.303 µM) versus controls (0.045 µM), revealing sustained oxidative stress across both attack and recovery phases. H_2_O_2_ in exhaled breath condensate showed good sensitivity for detecting oxidative stress, but its specificity was limited, as levels remained elevated after recovery and showed no correlation with lung function, while the absence of standardized collection methods and validated pediatric reference ranges further restricts its clinical utility [53]. Similarly, Xie et al. reported that children who maintained mid-to-high FeNO values (≥34.5 ppb) after clinical remission experienced more frequent relapses, underscoring FeNO as a predictor of future, more severe exacerbations. FeNO showed moderate sensitivity (69.2%) and high specificity (93.7%) for diagnosis, but its clinical applicability is restricted by limited standardization across age groups, measurement techniques, and comorbid allergic conditions [54]. Table 3 summarizes the clinical biomarkers of oxidative stress in childhood asthma.

At the cellular level, oxidative stress can self-amplify via a ROS-induced autophagy loop. While acute ROS exposure activates autophagy in airway epithelial and immune cells, chronic oxidative stress ultimately dysregulates the pathway, and the resulting defective autophagic flux further boosts ROS production and type-2 inflammation [55].

Given the central role of oxidative stress in driving persistent airway inflammation, barrier dysfunction, and airway remodeling, it stands to reason that this redox imbalance would also impact therapeutic effectiveness. Studies have demonstrated that oxidative stress not only perpetuates airway pathology but also impairs corticosteroid efficacy in asthma by reducing histone deacetylase 2 (HDAC2) activity, altering glucocorticoid receptor signaling, and activating pro-inflammatory pathways, all of which contribute to the emergence of steroid resistance in asthma [56,57].

## 7. Environmental & Lifestyle Modifiers of Pediatric Airway Redox Imbalance

Environmental pollutants and lifestyle factors together shape the oxidative balance in developing airways, influencing lung growth, inflammation, and the risk and severity of pediatric asthma [58,59,60,61,62]. The SEPAGES cohort study linked each interquartile-range increase in the oxidative potential of maternal personal PM_2.5_ exposure during pregnancy to a 2.3 mL decrease in infant functional residual capacity at six weeks, even after adjusting for concurrent NO_2_—evidence that combined PM_2.5_/NO_2_ exposures increase fetal oxidative stress and limit early lung growth [58]. Across the Barwon Infant Study, prenatal PM_2.5_ or NO_2_ exposure showed no association with infant lung function at four weeks or with maternal and cord-blood markers of inflammation or oxidative stress. However, in newborns carrying high-risk oxidative-stress genotypes, each interquartile-range rise in NO_2_ corresponded to a 5.3 mL reduction in functional residual capacity and a 0.46-turn increase in lung-clearance index, underscoring gene–environment synergy in shaping early lung development [6]. In a pooled cohort study of 1,188 term births from six U.S. cities, each 2-ppb rise in average O_3_ during the first two years of life was associated with roughly 30% higher odds of current asthma (odds ratio (OR): 1.31) and wheeze (OR: 1.30) at ages 4–6 years. However, this association was not observed at ages 8–9 years, suggesting that early-life O_3_ exposure, whether alone or in mixture with PM_2.5_ and NO_2_, chiefly triggers respiratory problems in early childhood [59].

Acute ambient insults impose an extra oxidative burden on infants. Drawing on prescription claims for more than 180,000 western-U.S. infants, Dhingra et al. found that each additional wildfire-smoke day, rich in particle-bound ROS, during weeks 0–12 (hazard ratio (HR): 1.094) and weeks 13–24 (HR: 1.108) of life significantly hastened the first fill of upper-respiratory medications, underscoring smoke-driven oxidative stress as a trigger for early-life respiratory exacerbations [63].

In a recent panel study of school-age children with asthma, each 2 °C drop in personal ambient temperature (within the 7–18 °C range) increased nasal MDA by 47–77%. Mediation analysis showed that this oxidative-stress marker explained 14–57% of the colder-temperature-related decline in Childhood Asthma Control Test scores, directly linking cold exposure, nasal MDA, and symptom worsening [5]. An emerging mechanistic layer associates pollution with redox-active metabolites via the airway microbiome. Air-pollution-driven dysbiosis of the airway microbiome generates oxidative metabolites that raise local ROS, activate NF-κB signaling (elevating IL-17 and allied cytokines), and thereby sustain the type-2-skewed inflammatory milieu characteristic of asthma [64].

Alongside these environmentally driven oxidative insults, excess adiposity functions as a lifestyle amplifier of the same redox pathways, further compromising the overall asthma burden. In 6- to 11-year-olds with asthma, a study found that obesity markedly amplifies oxidative stress: serum leptin, as well as MDA, and 8-isoprostane in exhaled-breath condensate, were all significantly higher in the obese-asthma group than in non-obese asthmatics and healthy controls, and each of these markers rose in proportion to body mass index (BMI) (MDA r = 0.48; 8-isoprostane r = 0.47). These oxidative-stress indices and the forced expiratory volume in 1 second (FEV_1_)/forced vital capacity (FVC) ratio were worst in children whose asthma remained uncontrolled, indicating that excess adiposity intensifies airway oxidative injury and compromises asthma control [60].

Conversely, in Portuguese schoolchildren, Rodrigues et al. reported that every one-point rise in the alternate Mediterranean-diet score reduced the adjusted odds of elevated FeNO (≥35 ppb)—a marker of nitrosative oxidative stress—by 23% in non-overweight children (OR: 0.77, 95% CI (confidence interval): 0.61–0.97), while the association was non-significant and numerically reversed among their overweight/obese peers (OR: 1.57, 95% Cl: 0.88–2.79) [61]. Moreover, in 70 children with asthma, six weeks of inspiratory-muscle training (IMT) (30% of maximal inspiratory pressure, seven days per week) significantly lowered systemic oxidative-stress load, periostin, and TGF-β levels compared with the control group (*p* < 0.05), while simultaneously boosting inspiratory and expiratory muscle strength and improving spirometric indices to values approaching those of healthy peers; no corresponding changes occurred in controls, confirming IMT as an effective adjunct therapy for dampening inflammation and oxidative stress in pediatric asthma [62].

Alongside these environmentally and lifestyle-driven oxidative insults, emerging evidence highlights that prenatal and early-life exposures beyond classical pollutants may exert comparable or even greater harm. According to Gambadauro et al.’s review, exposure to e-cigarette aerosols during pregnancy exerts profound adverse effects on fetal lung development, with oxidative stress and inflammation impairing epithelial maturation and alveolar formation. In the neonatal period, these insults persist, leading to sustained cytokine dysregulation, reduced antioxidant defenses, and long-term vulnerability to chronic respiratory diseases, such as asthma [65].

## 8. Genetic and Epigenetic Modifiers

Inherited genetic and epigenetic variations critically shape each child’s oxidative resilience and asthma susceptibility. Wu et al. found that the GSTP1 rs1695 (A > G), rs4891 (T > C), and CAT rs7943316 (A > T) variants each increased asthma risk across heterozygous, dominant, and allelic models, whereas the CAT rs769217 (C > T) allele was protective against childhood asthma. Haplotype analysis identified the GSTP1 GC combination as a risk haplotype (OR: 2.12, *p* = 0.025) and the CAT ATT haplotype as protective (OR: 0.45, *p* = 0.006), and MDR interaction modeling showed that rs1695 and rs7943316 together formed the strongest predictive model for asthma susceptibility [7].

In inner-city children with persistent atopic asthma, epigenome-wide profiling revealed 81 differentially methylated regions, 73 hypomethylated and 8 hypermethylated, adjacent to key T-lymphocyte and TH2-immunity genes (IL13, runt-related transcription factor 3 (RUNX3), TIGIT), even after adjusting for age, sex, race/ethnicity, and batch effects. Within asthmatic patients, 11 of these differentially methylated regions (DMRs) correlated with serum immunoglobulin E (IgE) levels and 16 with percent-predicted FEV_1_, and integrative LCMix analysis uncovered 2484 high-confidence methylation–expression pairs (including IL4, RUNX3, ST2), with pyrosequencing validation confirming asthma-associated methylation at RUNX3, IL4, CAT, kruppel-like factor 6 (KLF6), and NBR2 loci [66].

Ercan et al. demonstrated that the GSTP1 val/val genotype was independently associated with a 4.2-fold increased risk of more severe asthma in children (95% CI: 1.6–11.2; *p* = 0.004). Furthermore, children with this genotype exhibited higher systemic oxidative stress—reflected by elevated MDA and reduced GSH levels (*p* = 0.023 and *p* = 0.014, respectively) [67]. Similarly, Wang et al. found that the SOD2 (rs5746136) TT genotype, associated with impaired antioxidant defense, significantly increased asthma risk in children (adjusted OR = 2.78; 95% CI: 1.54–5.02). This genetic effect was further amplified under high phthalate exposure (MEHHP), with the asthma risk rising to OR = 3.32 (95% CI: 1.75–6.32), indicating a gene–environment interaction involving oxidative-stress-related pathways [68].

Additionally, based on Manti et al.’s review, oxidative stress during pregnancy and the neonatal period not only disrupts immune tolerance but also establishes peculiar epigenetic patterns that can persist through the activation of oxidative stress-related genes, thereby programming long-term susceptibility to atopic diseases. These mechanisms involve DNA methylation, histone modifications, and microRNA regulation, which influence Th-cell differentiation and immune polarization toward a Th2 phenotype, ultimately linking maternal and neonatal oxidative stress to the heritable epigenetic imprinting of atopy [69].

## 9. Therapeutic Strategies

### 9.1. Reducing Exposure to Environmental Oxidants—Lifestyle Factors

Reducing exposure to environmental oxidants remains fundamental in the prevention and management of oxidative stress in childhood asthma. As previously discussed, a variety of exogenous oxidants, including air pollutants (PM_2.5_, nitrogen dioxide, ozone), tobacco smoke, wildfire smoke, e-cigarette aerosols, and indoor pollutants, can trigger and sustain airway inflammation by increasing ROS [5,59,65]. Early-life exposure to such pollutants is linked to lower lung function, increased asthma risk, and more frequent exacerbations, with prenatal and early postnatal exposures exerting long-lasting effects, especially in genetically susceptible children [6,7]. Public health interventions, such as clean air policies, smoke-free environments, and parental education, are therefore crucial for minimizing children’s exposure and protecting respiratory health.

Likewise, as previously noted, lifestyle factors critically modulate oxidative stress and asthma outcomes. Excess adiposity significantly amplifies airway oxidative injury, with obese children demonstrating higher oxidative stress markers (e.g., MDA and 8-isoprostane) and poorer asthma control [60]. Conversely, structured exercise interventions, such as inspiratory muscle training, have been shown to lower systemic oxidative stress, reduce inflammation, and improve muscle strength and lung function [62]. According to a study by Onur et al., participation in a structured physical exercise program in children with asthma significantly increased antioxidant enzyme activity (glutathione peroxidase (GSH-Px) and SOD) and reduced oxidative stress markers, thereby improving lung function [70]. These findings further support the importance of holistic lifestyle changes, such as weight management and regular physical activity, as essential adjuncts to conventional therapy in optimizing redox balance and asthma control.

### 9.2. Dietary Antioxidants

Dietary modulation of oxidative stress is pivotal, as specific nutrients exert antioxidant effects that neutralize ROS and limit cellular injury. Diets rich in fruits, vegetables, whole grains, and unsaturated fats, exemplified by the Mediterranean Diet, provide key antioxidants such as vitamins C and E, beta-carotene (β-carotene), along with polyphenols, flavonoids, and carotenoids [42]. Adherence to this dietary pattern is associated with a protective effect against childhood asthma [71]. A randomized controlled trial found that an antioxidant-rich diet supplementation, specifically tomato juice when combined with vitamin C, can significantly improve asthma control, quality of life, and increase serum β-carotene levels in pediatric patients with mild to moderate persistent asthma; however, it showed no effect on pulmonary function tests or inhaled corticosteroid dosage [72]. Adequate antioxidant vitamin intake is correlated with improved pulmonary function and reduced wheezing, whereas deficiencies in vitamins C, E, and β-carotene are linked to poorer spirometry outcomes and increased asthma severity in children [73,74,75,76].

Vitamin C, a potent water-soluble antioxidant, has been shown in rabbit models to scavenge ROS intra- and extracellularly. Particularly, it demonstrated antioxidant properties in vivo by significantly reducing lipid peroxidation and preserving SOD activity during myocardial ischemia–reperfusion in rabbits. [77]. It may modulate asthma by lowering C-reactive protein (CRP) and inhibiting prostaglandins [78]. A cross-sectional study in children with persistent asthma showed that both zinc and vitamin C deficiencies were significantly associated with more severe asthma, reduced pulmonary function, and increased airway inflammation. Despite adequate dietary intake, all pediatric participants had zinc deficiency, and nearly 40% had vitamin C deficiency, suggesting enhanced metabolic utilization driven by elevated oxidative stress [75].

Vitamin E, primarily as α-tocopherol, protects lipid membranes from oxidative damage. The γ-tocotrienol isoform reduces allergen-induced airway inflammation by inhibiting NF-κB and activating Nrf2, with efficacy comparable to corticosteroids [79]. Tocopherol supplementation attenuates eosinophilic and neutrophilic inflammation [80], and its activity is potentiated by vitamin C-mediated regeneration [81]. Tocopherols also inhibit leukotriene synthesis in a plasma concentration-dependent manner [82] and have been associated with lower serum IgE levels and allergen sensitization [83]. Notably, plasma concentrations of vitamin E are significantly lower in both children and adults with asthma [84,85].

Β-carotene, a provitamin A carotenoid, functions as a lipid-soluble antioxidant capable of quenching singlet oxygen and inhibiting lipid peroxidation. It plays a critical role in preserving epithelial integrity and modulating inflammatory responses. Low serum β-carotene levels have been associated with increased oxidative DNA damage and heightened pulmonary inflammation [86]. Its deficiency, as a contributor to overall vitamin A insufficiency, has been linked to an elevated risk of asthma and respiratory morbidity [87,88].

Minerals such as zinc exert antioxidant activity by limiting •OH formation from H_2_O_2_ and simultaneously function as cofactors for SOD, enhancing the enzymatic defense against oxidative stress [47]. A recent meta-analysis revealed that circulating zinc levels were significantly inversely associated with the risk of childhood asthma, indicating that lower zinc concentrations are correlated with increased asthma severity and inflammation in pediatric patients. Furthermore, subgroup analysis showed that children diagnosed with asthma had significantly lower zinc levels compared to controls, reinforcing the protective role of adequate zinc status against asthma risk [89].

Polyphenols represent a structurally heterogeneous class of plant-derived secondary metabolites abundantly present in fruits, vegetables, tea, and grapes. They encompass several subclasses, including phenolic acids, flavonoids, catechins, tannins, lignans, stilbenes, and anthocyanidins, all of which exert significant antioxidant and anti-inflammatory effects [90]. Compelling preclinical data suggest that polyphenols attenuate respiratory inflammation, underscoring their potential as adjunctive therapeutic agents in asthma management [91]. Mechanistically, polyphenols scavenge reactive ROS, thereby mitigating oxidative stress and preserving pulmonary cellular integrity against injury induced by airborne pollutants and allergens [90]. Inflammatory cascades are modulated through the downregulation of key pro-inflammatory cytokines, including TNF-α, IL-6, and IL-1β, as well as the inhibition of the NF-κB signaling pathway, a central regulator of chronic airway inflammation [92]. Among polyphenols, flavonoids have been shown to reduce IgE titers and suppress airway inflammation in murine models of asthma. Moreover, compounds such as apigenin, fisetin, and luteolin selectively inhibit IL-4 production, further contributing to their immunomodulatory potential [93]. Polyphenols also benefit airway physiology by reducing mucus hypersecretion and promoting bronchial smooth muscle relaxation, thereby decreasing AHR [94].

### 9.3. Pharmacological Agents

N-acetylcysteine (NAC) acts as an antioxidant by neutralizing free radicals and boosting intracellular glutathione production. It increases Nrf2, a transcription factor for antioxidant enzyme synthesis [95,96]. NAC also reduces mucus viscosity by disrupting mucin disulfide bonds [97]. At high doses, it lowers the release of pro-inflammatory cytokines such as IL-9 and TNF-α [98]. In a mouse model of steroid-resistant asthma, NAC significantly reduced AHR and airway inflammation by decreasing neutrophil and eosinophil infiltration, as well as lowering IL-5 and IL-13 levels [99].

Nrf2 exerts a key protective role in asthma by upregulating antioxidant and cytoprotective genes, thereby reducing oxidative stress, limiting airway inflammation, eosinophilia, mucus hypersecretion, and AHR. Additionally, Nrf2 negatively regulates NF-κB signaling, further suppressing pro-inflammatory cytokine production, highlighting its potential as a therapeutic target in asthma and other inflammation-associated diseases [100].

Coenzyme Q10 (CoQ10), a key component of the mitochondrial electron transport chain, also functions as a crucial mitochondrial antioxidant by neutralizing free radicals and preventing the peroxidation of lipids and proteins [101]. A potential benefit of CoQ10 in asthma was observed in a study where supplementation with CoQ10 was linked to a reduction in the required corticosteroid dose [102]. In the study by Du et al., CoQ10 supplementation in a mouse model of allergic asthma significantly reduced airway eosinophilia, type 2 cytokines, IgE, and histamine levels, while upregulating Nrf2 expression and antioxidant defense genes, thereby attenuating airway inflammation, mucus hypersecretion, and oxidative stress injury [103].

Treatment with the p38 mitogen-activated protein kinase (MARK) inhibitor, especially SB239063, reduced airway resistance, improved lung compliance, and decreased IL-13 levels in ozone-exposed allergic asthma. Combined with α-tocopherol, it synergistically suppressed neutrophilic inflammation and C-X-C motif chemokine ligand 1 (CXCL-1) expression [104]. Inhibition of phosphoinositide 3-kinase (PI3K) attenuates airway inflammation, mucus hypersecretion, and immune cell recruitment, and is considered a promising therapeutic target for oxidative stress-driven asthma phenotypes [105].

**Figure 2:**
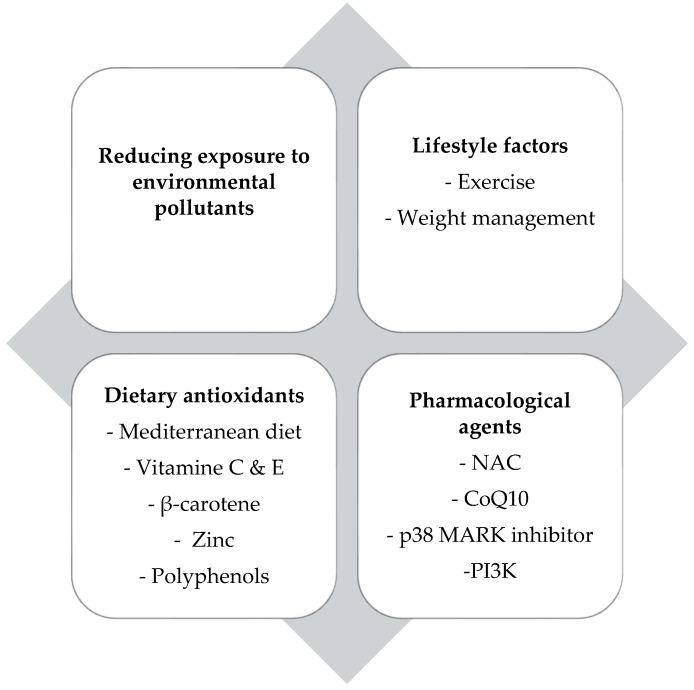
Overview of therapeutic strategies that mitigate oxidative stress in childhood asthma. NAC: N-acetylcysteine, CoQ10: Coenzyme Q10, MARK: mitogen-activated protein kinase, PI3K: phosphoinositide 3-kinase.

Figure 2 summarizes the therapeutic strategies targeting oxidative stress in childhood asthma.

## 10. Future Considerations

Future research should prioritize well-designed clinical trials to confirm the efficacy and safety of antioxidant therapies in childhood asthma. Clarifying how genetic and environmental factors, such as antioxidant gene variants and prenatal exposures, interact will advance precision medicine. Non-invasive redox biomarkers (e.g., exhaled-breath condensate, proteomics) may improve early diagnosis, risk assessment, and monitoring. The airway and gut microbiome also show promise as therapeutic targets for redox balance. Importantly, personalizing prevention and treatment using individual redox and genetic profiles should be a key goal. Ultimately, combining lifestyle change, dietary antioxidants, and pharmacological agents may offer the most effective management for pediatric asthma.

## 11. Conclusions

Oxidative stress is a central mechanism in the development and exacerbation of childhood asthma, leading to airway barrier dysfunction, increased inflammation, and reduced steroid effectiveness. Both internal and external sources of ROS and RNS contribute to disease severity and poor control, especially in genetically vulnerable children or those with high environmental exposures. While antioxidant defenses exist, they may be inadequate in high-risk groups. Strategies that target oxidative stress, including environmental interventions, dietary optimization, physical activity, and pharmacological approaches, hold promise for improving outcomes. Personalized therapy based on redox status and genetic risk may further enhance asthma management in children. Ongoing translational research remains imperative to substantiate these approaches and to enable the personalization of therapy through individual redox status and genetic susceptibility, ultimately transforming the care and prognosis of pediatric asthma.

## Figures and Tables

**Table 1:** Main sources and effects of oxidative stress in asthma.

Source	Examples/Key Molecules	Main Effects in Asthma
Endogenous	Mitochondria, NADPH oxidases (NOX2, NOX4), immune cells [4,11,12,13]	Increase of ROS/RNS, airway inflammation, tissue remodeling
Exogenous	Air pollution (PM_2.5_, NO_2_, O_3_), tobacco smoke, allergens [4]	Initiation of oxidative reactions, worsened control
Genetic Factors	GSTP1, CAT gene polymorphisms [7]	Modify susceptibility, affect the antioxidant response
Microbiome (Dysbiosis)	Altered gut–lung axis, Reduction of SCFAs production [8]	Enhances type 2 inflammation, increases ROS

NADPH: nicotinamide adenine dinucleotide phosphate, ROS: reactive oxygen species, RNS: reactive nitrogen species, PM_2.5_: particulate matter, NO_2_: nitrogen dioxide, O_3_: ozone, GSTP1: glutathione s-transferase Pi-1, CAT: catalase, SCFAs: short-chain fatty acids.

**Table 3:** Clinical biomarkers of oxidative stress in asthma.

Biomarker	Measurement/Source	Clinical Significance
8-Isoprostane [52]	Exhaled breath	Increased in children with asthma, marker of exacerbation risk
H_2_O_2_ [53]	Exhaled breath condensate	Elevated during exacerbation and recovery
FeNO [54]	Exhaled breath	High values predict more frequent relapses
MDA [5]	Nasal/serum samples	Linked to symptom worsening, cold exposure

H_2_O_2_: hydrogen peroxide, FeNO: Fractional exhaled nitric oxide, MDA: malondialdehyde.

## Data Availability

Data sharing is not applicable to this article.
